# Comprehensive review on gene mutations contributing to dilated cardiomyopathy

**DOI:** 10.3389/fcvm.2023.1296389

**Published:** 2023-12-01

**Authors:** Shipeng Wang, Zhiyu Zhang, Jiahuan He, Junqian Liu, Xia Guo, Haoxuan Chu, Hanchi Xu, Yushi Wang

**Affiliations:** ^1^ Department of Cardiovascular Medicine, The First Hospital of Jilin University, Changchun, China; ^2^ Department of Cardiovascular Medicine, The Second People's Hospital of Yibin, Yibin, China

**Keywords:** dilated cardiomyopathy, DCM, gene mutations, molecular mechanism, therapy, targeted therapy, genetic testing

## Abstract

Dilated cardiomyopathy (DCM) is one of the most common primary myocardial diseases. However, to this day, it remains an enigmatic cardiovascular disease (CVD) characterized by ventricular dilatation, which leads to myocardial contractile dysfunction. It is the most common cause of chronic congestive heart failure and the most frequent indication for heart transplantation in young individuals. Genetics and various other factors play significant roles in the progression of dilated cardiomyopathy, and variants in more than 50 genes have been associated with the disease. However, the etiology of a large number of cases remains elusive. Numerous studies have been conducted on the genetic causes of dilated cardiomyopathy. These genetic studies suggest that mutations in genes for fibronectin, cytoskeletal proteins, and myosin in cardiomyocytes play a key role in the development of DCM. In this review, we provide a comprehensive description of the genetic basis, mechanisms, and research advances in genes that have been strongly associated with DCM based on evidence-based medicine. We also emphasize the important role of gene sequencing in therapy for potential early diagnosis and improved clinical management of DCM.

## 1. Introduction

Dilated cardiomyopathy (DCM) is characterized by the enlargement of the left ventricle (LV) and global or regional systolic dysfunction, which cannot be solely attributed to abnormal loading conditions such as hypertension, valve disease, congenital heart disease, or coronary artery disease (1, 2). It is considered one of the main causes of heart failure with reduced ejection fraction (HFrEF) worldwide. Genetic defects are the primary cause of DCM, with approximately 30%–35% of idiopathic cardiomyopathy being attributed to genetic defects. The majority of genes associated with genetic DCM exhibit autosomal dominant transmission, while a minority follow an autosomal recessive, X-linked, or mitochondrial inheritance pattern (3). There are about 60 genes associated with DCM, and evidence-based medicine has identified 12 genes that are highly associated with the condition (4, 5). Familial DCM can be inherited as a recessive or *X*-linked trait, although autosomal dominant inheritance is the most common (6). Non-genetic factors also play a significant role in DCM, and there is overlap between genetic and non-genetic causes (7). The objective of this review is to provide a comprehensive summary of the genes that are clearly associated with DCM and to recommend sequencing of known cardiomyopathy genes for all DCM patients. It is important to note that genetic causes of DCM cannot be ruled out in patients with no family history, as *de novo* mutations may be responsible for the development of the condition (4, 8–10). The standard treatment for heart failure resulting from DCM involves drug therapy, such as loop diuretics, angiotensin-converting enzyme inhibitors (ACEIs), beta blockers, and sodium-glucose cotransporter 2 inhibitors (SGLT2i), as well as cardiac resynchronization therapy (CRT) (11, 12). However, current medications do not halt myocardial degeneration, and heart transplantation remains the only option for patients in the advanced stages of heart failure (13, 14). Nevertheless, the precise pathological mechanisms responsible for variations in disease susceptibility and phenotypic expression, including the risk of heart failure (HF) or sudden cardiac death (SCD), remain elusive (15, 16). As the genetic dimension of DCM becomes better understood, gene therapy is emerging as a promising treatment strategy for DCM (17).

## 2. Epidemiology

Reliable epidemiological data on cardiomyopathy is primarily sourced from developed countries, where the accuracy of prevalence data is dependent on the use of established diagnostic evaluations and criteria. However, there is still a dearth of epidemiological data concerning DCM in Asia (7). The Olmsted County study concluded that the prevalence of idiopathic DCM was 1 in 2,500. However, it is possible that the incidence and prevalence of DCM may be greatly underestimated due to various biases, such as misclassification and missing or incomplete data. Recent studies suggest that the prevalence of asymptomatic idiopathic DCM may be equal to or greater than 1 in 250 (3, 18, 19). The incidence of DCM is slightly higher in males than in females, with an average sex ratio of 1.7:1 for males with hereditary DCM and 2.5:1 for females with non-hereditary DCM. The long-term prognosis for females is better than that for males (20–22). However, these data are based on estimates, and a formal, population-based epidemiological study is still needed to determine the true prevalence and incidence of DCM. Researches have shown that 26% of children with dilated cardiomyopathy experience either death or the need for a heart transplant within one year of diagnosis, with an additional 1% per year thereafter (23). DCM is also the most common cause of chronic congestive heart failure and sudden cardiac death in individuals aged 20–60, as well as the leading cause of heart transplants (24).

## 3. Clinical manifestation

Symptoms of DCM, such as dyspnea, fatigue, dizziness, syncope, and edema, may intermittently manifest in some patients during the early stages of DCM. However, these symptoms become more pronounced as the disease progresses to its severe stage (25). Uncommon yet significant signs and symptoms like abnormal skin pigmentation, skeletal myopathy, and neurosensory disorders (e.g., deafness, blindness) may indicate a specific form of multisystem disease or a unique DCM genotype. These symptoms are considered “red flags” for DCM diagnosis (26).

## 4. Etiology

The etiology of dilated cardiomyopathy can be categorized into genetic causes, which lead to primary dilated cardiomyopathy, and acquired factors resulting in secondary dilated cardiomyopathy. It is imperative for clinicians to rule out secondary causes prior to confirming a diagnosis of “idiopathic DCM”, as there might be potential reversible causes (27, 28). Single gene mutations account for 25%–50% of all DCM cases. Genes linked to DCMs can be categorized into various groups, which encompass those encoding nuclear envelope proteins, sarcomere proteins, structural proteins, ion channels, and proteins yet to be classified (29). Jan Haas and his team identified a notably higher mutation rate in familial instances compared to sporadic ones. Even when genetic investigations fail to elucidate a familial disease, alternative mechanisms like epigenetic modifications—including microRNAs, histone modifications, and DNA methylation—should be considered (30). There are also certain genetic mutations that can indirectly lead to DCM by affecting the stability of crucial cardiac structures. For instance, The *Z*-disc in cardiac myocytes is a crucial region where numerous proteins interact within the *Z*-disc to facilitate force transmission and intracellular signaling in both the heart and skeletal muscles (31, 32). Kindlin-2 collaborates with α-actinin-2 and β1 integrin to preserve the structural integrity of the *Z*-disc in cardiac muscle tissue (33). The elimination of Kindlin-2 in murine models results in the disruption of the *Z*-disc structure, subsequently causing cardiac malfunction (34, 35). Increasingly, research studies are documenting the existence of multiple potentially causative mutations in patients diagnosed with Dilated Cardiomyopathy (DCM). While a significant number of these are likely silent variants, an emerging model of oligogenic inheritance—a disease provoked by a limited number of mutations across multiple genes—is being recognized (36). These acquired factors encompass infections, excess alcohol consumption, exposure to toxins, cancer therapies, endocrine disorders, pregnancy, tachyarrhythmias, and immune-mediated diseases (37).

## 5. Genes strongly associated with DCM

**Figure 1:**
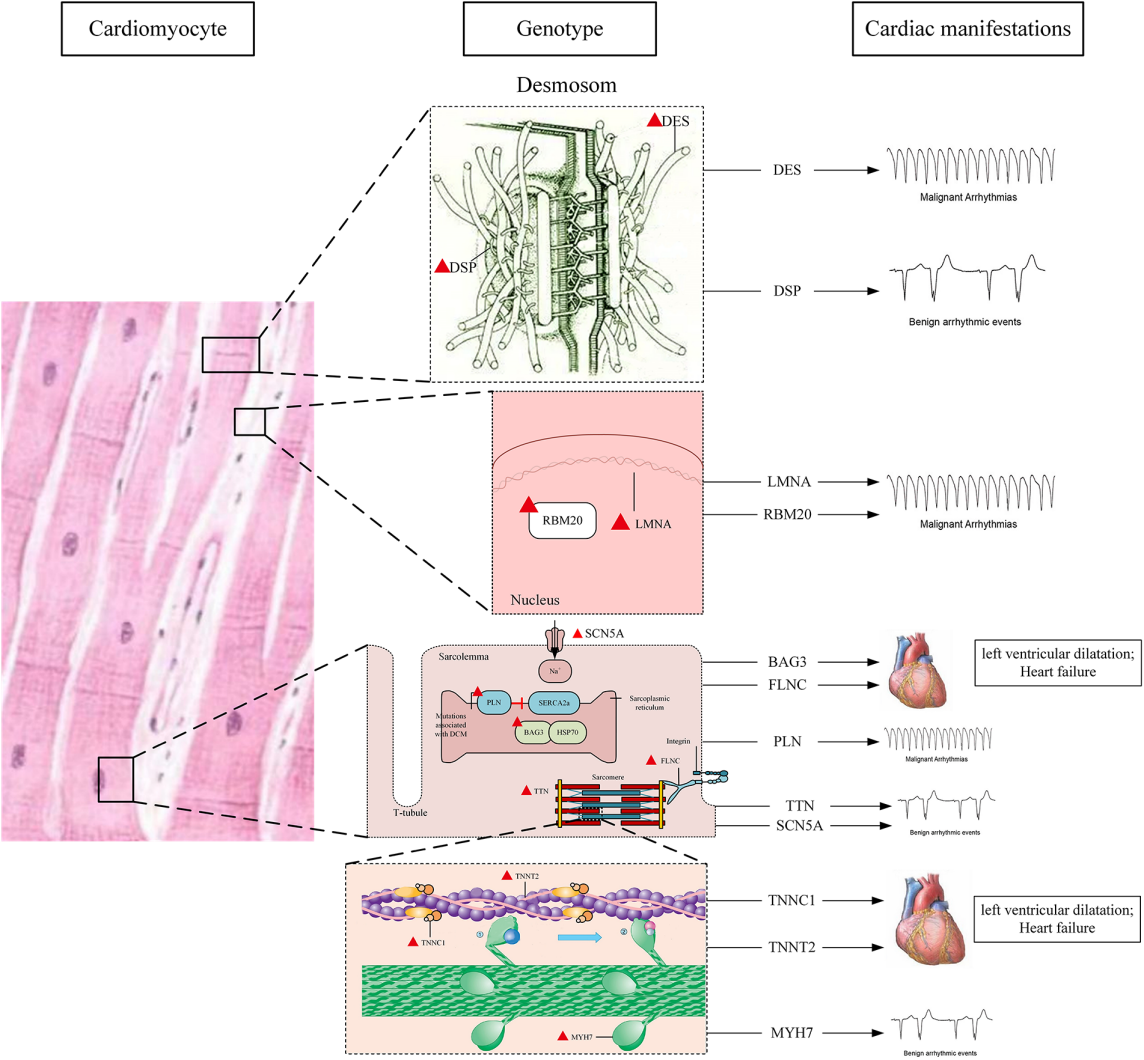
Location and major cardiac manifestations of DCM-related genes.

The location and major cardiac manifestations of DCM related genes are shown in Figure 1.

### 5.1. TTN

The Titin Gene (TTN) is one of the largest genes found in humans, encoding the protein titin. With a molecular weight of 3,816 kDa, titin is the largest known polypeptide and a giant muscle protein that spans half of the myotome from the *Z* line to the *M* line. It serves as the fundamental structural and functional unit of striated muscle, with its presence in both the heart and skeletal muscle (25, 38, 39). The TTN gene expresses two major isoforms in the heart, namely N2B and N2BA. These isoforms contain four distinct regions referred to as *z*, *i*, *a*, and *m* lines (40). Titin proteins not only provide structural, mechanical, and regulatory support but also play a pivotal role in the passive and active contractility of skeletal muscles (41). Truncation mutations of TTN (TTNtv) are most prevalent in DCM, contributing to approximately 25% of familial DCM cases and 18% of sporadic DCM cases (40, 42). A significant number of TTN mutations, responsible for DCM, are heterozygous truncation variants of TTNtv. These include frame-shift mutations, nonsense mutations, and critical splice-site mutations, which are predominantly overexpressed in the A-band region of the titin protein. Additionally, a minor percentage of DCM cases may also be attributed to missense mutations in titin (43–45). The clinical implications of TTNtv are largely contingent on exon expression and the location of the mutation. TTNtv is predominantly located in the A-band, with the severity of the disease increasing as the mutation approaches the C-terminal, indicating a location dependence (43, 44). The exact mechanism through which titin truncation mutations induce cardiac phenotypes remains uncertain. Several mechanisms have been proposed to elucidate TTNtv-induced DCM, including haploinsufficiency, poison peptide/dominant negative mechanism, disruption of cardiac metabolism and signaling, and loss of function. However, these mechanisms warrant further validation through subsequent studies (43, 46–48). The mutation in TTNtv instigates a metabolic transition in the cardiac system from fatty acids to glycolysis (49). However, a chronical elevation in glycolytic intermediates and branched-chain amino acids may trigger the activation of the serine/threonine protein kinase mTOR complex 1 (mTORC1) signaling pathway. This activation subsequently stimulates inefficient protein synthesis cycles and suppresses autophagy (47, 50). TTNtv is characterized by frequent arrhythmias, mainly atrial fibrillation and ventricular arrhythmias (51, 52). Patients with DCM who are TTNtv-positive have a significantly heightened risk of persistent ventricular tachycardia compared to TTNtv-negative patients (44). Studies suggest that in a certain percentage of patients, TTNtv may interact synergistically with other factors (e.g., cardiotoxic chemotherapy, pregnancy), implying that treating these exacerbating factors could lead to substantial recovery (47). As our understanding of gene mutations improves, the potential for genetic engineering-based therapies becomes apparent. Techniques such as reverse-mediated exon skipping, targeted therapy, and genome editing strategies could provide promising therapeutic opportunities (53–55).

### 5.2. LMNA

The Lamin A and Lamin C Gene (LMNA) is comprised of 2,400 base pairs of DNA and 12 exons. Lamins A and C, intermediate filamentous nuclear envelope proteins, are encoded by the LMNA gene. This nuclear membrane protein is situated in the inner part of the nuclear membrane and is generally expressed in differentiated cells (56). The LMNA gene is the second most frequently mutated gene in DCM。The occurrence rate of Lamin A/C variations in patients diagnosed with DCM is approximately 6% (57, 58). Patients with pathogenic LMNA mutations exhibit a high rate of sudden cardiac death due to malignant arrhythmias and a poor prognosis (59, 60). In laminopathy, frameshift mutation is often associated with heart disease, splice site mutation is an independent risk factor for sudden cardiac death, and non-missense mutations (deletions/truncations or mutations affecting splicing) are major independent risk factors for malignant ventricular arrhythmias (MVAs) (61). The exact pathogenesis of DCM caused by LMNA mutation remains unclear, but three hypotheses—mechanical, gene expression, and cytotoxicity—have been proposed to explain the cardiac dysfunction associated with it. The mechanical hypothesis suggests that the destruction of the nuclear layer increases nuclear fragility and sensitivity to mechanical stress, making myocardial tissue more susceptible to pathological effects (62). One proposed mechanism views the nucleus as a mechanosensor that modulates gene expression in response to mechanical perturbations. Consequently, external forces transmitted via the cytoskeleton induce nuclear deformation. Simultaneously, contingent on lamin composition, there can be an impact on its transcriptional activity by altering chromatin's organization and positioning. Concurrently, any modifications in chromatin's structure and organization can potentially influence the nucleus's mechanical attributes (63). Given that the nuclear lamina possesses a higher degree of stiffness compared to the nuclear membrane, it serves to shield the latter from substantial mechanical forces. This buffering capability can notably influence the stretch response of the nuclear membrane, subsequently altering the distribution and organization of membrane-bound proteins. The force-induced expansion of nuclear membranes could potentially represent another mechanism activated by disrupted mechanotransduction (64). Studies indicate that decoupling the mechanical forces of the nuclear/nuclear skeleton and cytoskeletal transduction can significantly extend the lifespan of LMNA deficient mice (65). The gene expression hypothesis posits that defective lamins impede signal transduction and chromatin organization, thereby altering signal transduction, a key driver of LMNA-associated dilated cardiomyopathy. This directly affects and disrupts gene transcription and other intracellular signaling pathways, significantly increasing myocardial fibrosis and leading to left ventricular dysfunction and heart failure (66, 67). The cytotoxic hypothesis suggests that mutated prelamin A protein, also known as presenilin, contributes to the disease by disrupting nuclear morphology, heterochromatin distribution, and DNA damage repair pathways, leading to premature aging (28, 62). Due to the risk of sudden cardiac death in LMNA associated DCM being linked with heart block and bradycardia, the use of Implantable Cardioverter Defibrillators (ICDs) has been recommended for all indications (68). Various other pathways downstream of the LMNA gene have also been explored as potential therapeutic pathways, such as the use of rapamycin/rapalog to inhibit mTOR and MEK1/2 kinase pathway inhibitors, the inhibition of the activation of brominated domain protein 4 (BRD4), and the destruction of LINC complex protein SUN1 to inhibit LMNA mutations (65, 69–71). However, no specific and effective treatment is presently available.

### 5.3. DSP

Desmoplakin (DSP), encoded by the DSP gene, is a major component of desmosomes and is highly abundant in myocardial tissue. The DSP protein exhibits a tripartite structure, which includes a spherical N-terminal patch domain, a central *α*-helical rod domain, and a C-terminal tail domain. The DSP gene, located on chromosome 6p24.3, undergoes alternative splicing to produce three subtypes: DSP-I (long), DSP-IA (intermediate), and DSP-II (short). DSP-I is the primary cardiac subtype and plays a crucial role in intercellular adhesion within cardiomyocytes (72, 73). Gene-targeting studies in mice have revealed that mice with ablated DSP genes die during early embryonic development (74). In an assessment of genes associated with DCM, DSP emerged as the highest-scoring gene. However, the arrhythmia phenotype's potential to complicate the interpretation of experimental data led to questions about the trial score. Consequently, the DSP gene was identified as a strong contributor to DCM, rather than the definitive cause (4). Some research suggests that DSP mutations are unique to adult DCM (75). Palmoplantar keratoderma may serve as an early clinical symptom of DCM associated with a DSP mutation (76). Carriers of DSP variants exhibit a higher rate of arrhythmia events, similar to those with LAMA variants, even in the absence of significant left ventricular dysfunction or dilation (77). Current research has also classified desmoprotein cardiomyopathy as an arrhythmic cardiomyopathy triggered by DSP mutations. This condition is characterized by paroxysmal myocardial injury, left ventricular fibrosis preceding systolic dysfunction, and a high incidence of ventricular arrhythmia (73, 78). Typical electrocardiogram (ECG) abnormalities include limb lead QRS depression (peak <0.5 mV) and lateral or inferior lead *T* wave inversion (73, 79). In arrhythmogenic cardiomyopathy (ACM) caused by DSP variation, cardiomyocytes release a large number of inflammatory cytokines and chemotactic molecules (80). Histological analysis of left ventricular myocaroma in DSP patients has also revealed inflammatory infiltration and scarring (81). Consequently, inflammation is considered a key feature of the disease, and regulating inflammatory signaling pathways may present a new therapeutic target for desmosome-mediated cardiomyopathy.

### 5.4. DES

DES encodes the primary intermediate filament (IF) protein, desmin, in human heart and skeletal muscle (82). Desmin serves as a structural component of the extramuscular cytoskeleton, which forms a three-dimensional scaffold around the Z disk of the myofibril. This structure connects adjacent myofibrils and the myofibril apparatus to the nucleus, submuscular cytoskeleton, and cytoplasmic organelles such as mitochondria (83). Desmin proteins exhibit a tripartite structure, comprising a conserved central rod domain flanked by non-alpha helical head and tail domains. The central rod domain consists of four helical segments (2A, 1B, 12A, and 2B) separated by three short polypeptide junctions (L1, L1, and L2) (84). There are over 73 different IF proteins, with Desmin being the most abundantly expressed IF protein in muscle-specific tissues and cardiomyocytes (85). IFs perform numerous tissue-specific functions, including providing mechanical support to cells and regulating intracellular tissues, stress responses, cell growth, proliferation, migration, and death (84–86). In patients with DCM, the incidence of desmin gene mutations is less than 1.6% (87). Most DES mutations are missense mutations within the central domain; nonsense, insertion, deletion, or combination insertion and deletion mutations are rare (88). Approximately 74% of DES mutation carriers exhibit cardiac symptoms, and about 50% develop cardiomyopathy, with dilated cardiomyopathy (DCM) being the most common type (89). Researches involving mice with DES gene knockout have demonstrated that desmin deficiency not only impacts heart structure but is also associated with severe abnormalities in myocardial metabolism of glucose, fatty acids, and amino acids (90, 91). Therefore, it may be prudent to avoid drugs that could potentially exacerbate mitochondrial function in patients with desmin deficiency (92). The α-crystallin Β-chain (αB-crystallin), encoded by Desmin and CRYAB, has a potential compensatory interaction in cardiac protection. Overexpression of heart-specific αB-crystallin improves mitochondrial dysfunction in desmin-deficient mouse models, suggesting a potential new treatment approach (93).

### 5.5. MYH7

The MYH7 gene, encoding the cardiac beta-myosin heavy chain, is situated on chromosome 14q11.2-q13. This gene consists of 40 exons that produce a MYH7 protein containing 1,935 amino acids. This protein is primarily expressed in ventricular muscle and type 1 skeletal muscle fibers, and it is a significant component of human ventricular myosin. The MYH7 protein plays a crucial role in the energy supply for cardiomyocytes and in maintaining the Ca2+ concentration inside and outside these cells (94, 95). MYH7 mutations account for 1%–5.3% of DCM cases (96). These mutations are predominantly missense variants, inherited in a chromosomal dominant pattern, with high penetrance in families and a relatively high proportion among children (95, 97). Mutations in the MYH7 gene can damage the integrity of the sarcomere structure or function, affecting the contraction of the heart muscle (95). Atrial fibrillation and atrial fibrosis are considered early clinical manifestations of MYH7-related cardiomyopathy, which may provide valuable insights for disease diagnosis (98). It has been reported that combined mutations in MYH7 and TNNT2, MYH7 and LAMA4, or MYH7 and TPM1 can result in severe DCM (99–101). These findings highlight the importance of comprehensive screening of DCM-related genes, even after identifying a single disease-causing mutation. Studies have shown that telomere length in mice can offer protection against heart disease in humans. Mutations in proteins that are critical to cardiomyocyte function, such as MYH7, TTN, and MYBPC3, lead to shorter telomeres. Consequently, significant telomere shortening can serve as a biomarker for premature aging of cardiomyocytes in hereditary Hypertrophic Cardiomyopathy (HCM) and DCM (102, 103). The Telomere Repeat Binding Factor 2 (TRF2) has been demonstrated to prevent telomere attrition, thereby improving cell morphological defects, activation of DNA damage response, and premature cell death (104).

### 5.6. BAG3

Bcl2-associated athanogene 3 (BAG3) codes for an anti-apoptotic protein located on the *Z* disc of myotomes. As a member of the anti-apoptotic BAG protein family, BAG3 is abundantly expressed in the heart, skeletal muscle, various types of tumor cells, as well as in the brain and peripheral nervous system (105, 106). BAG3 plays vital roles in anti-apoptosis, protein homeostasis maintenance, mitochondrial stability regulation, myocardial contraction regulation, and arrhythmia reduction (107, 108). The multifunctionality of BAG3 in cardiomyocytes is attributed to the presence of multiple functional domains, including the WW domain, the IPV (Ile-Pro-Val) motif, the proline-rich motif, and the BAG domain (109). However, studies indicate that all known or probable pathogenic variants impact at least the WW domain, IPV domain, or BAG domain, with none of the missense pathogenic or possibly pathogenic variants affecting the proline-rich motifs. These three protein domains play significant roles in BAG3's function in the heart (108). Large multicenter cohort studies reveal that DCM resulting from BAG3 mutations is characterized by early-onset in most patients, a high risk of progressing to end-stage heart failure, and a worse prognosis in males (110). BAG3, an anti-apoptotic constituent of the BAG protein family, possesses an inhibitory function against apoptosis. A notable surge in apoptosis was observed in mice lacking BAG3 (111). As a cochaperone protein, BAG3 interacts with ATP-dependent high molecular weight heat shock proteins and ATP-independent small heat shock proteins (sHSPs) in large, functionally different multichaperone protein complexes (112). And after BAG3 is lost, the affected sHSPs levels are reduced due to protein instability (112). BAG3-mediated macrophage recruitment can maintain protein homeostasis, autophagic flux was suppressed in BAG3-deficient hearts, which might result in misfolded protein aggregates (113). Consequently, there is an increase in the content of insoluble proteins, which accelerates the process of cellular senescence. MicroRNAs (miRNAs) are small non-coding RNAs (20–25 nucleotides) that function as epigenetic regulators in cardiovascular system development and physiology. Dysregulation of their expression is directly associated with the pathophysiology of numerous cardiovascular diseases (114, 115). Studies demonstrate that the co-expression of miR-154-5p and miR-182-5p holds diagnostic value in DCM of BAG3 mutation carriers (116). The transcriptional adaptation of gene expression triggered by harmful gene mutations, known as genetic compensation, has been observed in BAG3-knockout zebrafish to protect against heart and skeletal muscle damage. This biological phenomenon may also be active in some human carriers of BAG3 mutations (117). Further investigation of the relevant molecular mechanisms may offer fresh insights for the development of therapeutic interventions.

### 5.7. FLNC

The filamin family comprises three isomers: filamin A (FLNA), filamin B (FLNB), and filamin C (FLNC) (118). FLNA and FLNB are commonly expressed, while FLNC is most prevalent in skeletal and cardiac muscle (119). FLNC plays a crucial role in the regulation of cellular mechanics, *Z*-disk arrangement and orientation, and intermyofibrillar connections in mammalian hearts (120, 121). Deficiency of FLNC in cardiomyocytes can lead to fetal death. Furthermore, adult mice deficient in FLNC develop rapid and fulminant DCM within two weeks (122). These studies underscore the significant role of FLNC in both developing and adult cardiomyocytes. A truncation mutation in FLNC (FLNCtv) is closely associated with DCM (123). Patients with FLNCtv often exhibit left ventricular dilatation with systolic dysfunction and myocardial fibrosis. Ventricular arrhythmias are prevalent, and families carrying these mutations have a high incidence of sudden cardiac death (124, 125). β-catenin (CTNNB1) has been identified as the downstream target of FLNC through co-immunoprecipitation and proteomic analysis. FLNC is unable to induce nuclear translocation of CTNNB1, which subsequently activates the platelet-derived growth factor receptor-α (PDGFRA) pathway. Inhibition of PDGFRA can partially reverse the pathological gene expression profile of FLNC patient-specific cardiocytes, cardiac insufficiency, and arrhythmia (126). Therefore, inhibition of this pathway presents a potential therapeutic approach for FLNC-associated cardiomyopathy.

### 5.8. PLN

Phospholamban (PLN) is a 6.1 kDa protein situated on the sarcoplasmic and endoplasmic reticulum (SR/ER) membrane. PLN is responsible for the encoding of a crucial regulatory protein associated with Ca2+ cycling. It serves as a principal mediator of beta-adrenergic effects, which subsequently leads to an augmentation of cardiac output (127). It is regulated by protein kinase-mediated phosphorylation and serves as an inhibitor of the sarcoplasmic/endoplasmic reticulum Ca2+ ATPase (SERCA2a). Homeostasis and cardiac contractility are achieved by reversibly inhibiting SERCA2a activity (128). The interaction between SERCA2a and PLN determines the rate of diastole and contraction of cardiomyocytes (129). Impaired SR function in diastolic and systolic Ca2+ circulation is a critical factor in cardiac cardiomyocyte failure (130). Among all known PLN mutants, the PLN-R14DEL mutation appears to be the most prevalent (131, 132). The mutation of the PLN gene is not a common cause of cardiomyopathy in our population, with a mutation occurrence rate of less than 1% (133, 134). A large multicentre study with long-term follow-up of PLN mutation carriers found that early ventricular arrhythmia and end-stage heart failure were common in PLN-R14DEL mutation carriers, resulting in a significant increase in cohort mortality (135). From the perspective of a cardiomyocyte, it is recognized that PLN R14del significantly affects its function. Research has shown that the R14del mutation results in the aggregation of PLN protein, an escalation in the activity of the unfolded protein response, dysregulation of calcium, as well as contractile and metabolic dysfunction (136). Despite the well-defined genetic etiology, the molecular mechanism driving the pathogenesis of PLN R14del-cardiomyopathy remains elusive (137). Low-voltage electrocardiograms are more common in women carrying PLN mutations, but their prognostic value is higher in men (138). It has been proposed that eplerenone treatment can prevent or slow disease progression in presymptomatic PLN mutation carriers. Further multicentre randomized double-blind trials are being conducted to confirm this (139). As our understanding of the PLN-R14del mutation mechanism continues to improve, precision medicine, including gene editing and targeted gene therapy, may represent a new direction for future treatment (136, 140, 141). However, most current research remains in the animal testing phase, and it is unclear whether these findings will be applicable to human patients (142).

### 5.9. RBM20

The RNA-binding motif protein 20 (RBM20) primarily functions as a splicing regulator, predominantly expressed in the heart and skeletal muscle, where it orchestrates both constitutive splicing and alternative splicing of pre-messenger RNA (143, 144). RBM20 gene mutations, which predominantly manifest as missense mutations that alter conserved residues, are a leading cause of DCM (145, 146). This mutations account for approximately 3% of all DCM cases (143).

The pathophysiology of these RBM20 mutations stems from a combination of functional loss and pathogenic functional gain (147). Of the genes regulated by RBM20, TTN is the most significant (146, 148). Diminished RBM20 activity also results in the altered expression of protein subtypes that sustain muscle structure and cardiac function, such as CAMKIIδ, LDB3, and CACNA1C. These alterations can induce changes in biomechanics, electrical activity, and signal transduction, potentially leading to cardiomyopathy, fibrosis, arrhythmia, and sudden death (149, 150). Patients with DCM who carry RBM20 mutations often exhibit impaired cardiac function and are susceptible to atrial fibrillation, ventricular arrhythmia, and sudden cardiac death (151). All-trans retinoic acid (ATRA) has been identified as a potential regulator of RBM20, with studies showing that ATRA can increase RBM20 expression and partially restore the *in vitro* DCM phenotype. Therefore, pharmacological upregulation of RBM20 expression could be a promising therapeutic strategy for DCM patients with heterozygous RBM20 mutations (152). Most RBM20 mutations are clustered in an arginine/serine (RS) -rich domain, suggesting that precision gene editing using adenine base editing (ABE) and primer editing (PE) might offer potential treatments (147).

### 5.10. SCN5A

The sodium channel family is comprised of nine genes (SCN1A-SCN5A, SCN7A-SCN11A). Among these, the SCN5A gene, located on human chromosome 3p22, encodes the cardiac sodium channel pore-forming *α* subunit Nav1.5. SCN5A/Nav1.5 is predominantly expressed in the atrial and ventricular myocardium, His bundle, bundle branch, and Purkinje fibers (153). The prevalence of SCN5A-mediated cases in patients with dilated cardiomyopathy (DCM) is approximately 2% (154). Frameshift mutations in SCN5A can result in a loss of function of the heart's sodium channels (155). The molecular pathway through which these mutations cause ventricular dilation and dysfunction is yet to be fully understood (156). Mutations in SCN5A may interfere with the interaction between Nav1.5 and other components of the complex, leading to structural deformities and contractile damage. Two Nav1.5 mutations (R222Q and R225W) in the voltage sensor domain (VSD), situated in the voltage-gated ion channel, are hypothesized to generate gated hole currents that may be linked with arrhythmia and ventricular dilation in humans (157). Occult myocardial injury may also result from the impaired function of the mutated SCN5A immune sensor (158). DCM typically exhibits age-dependent penetrance, with the phenotype becoming more pronounced with age (159). Clinically, it often manifests as severe arrhythmias (including atrial fibrillation and ventricular tachycardia) and conduction block (154, 160). The initiation of sodium channel blockers can prevent significant morbidity and mortality (161). Research has revealed that the mRNA stabilizing protein HuR protects SCN3A by binding to 5'-UTR mRNA, preventing its decay. The risk of arrhythmia can be reduced by enhancing mRNA stability to preserve decreased SCN5A expression (162).

### 5.11. TNNC1, TNNT2

The TNNC1 gene (3p21.1) encodes cardiac troponin C (cTnC) in heart tissue, while the TNNC2 gene (1q32.1) encodes cardiac troponin T (cTnT) (163). The trimer filament Tn complex, involved in muscle contraction, is formed by the combination of cTnT, cTnI, and cTnC (164). Point mutations on TNNC1 can alter the function of cTnC in two ways: by changing its binding affinity for Ca2+ or by modifying the interaction of cTnC with its binding partner (165). Troponin complex mutations are present in approximately 6% of familial DCM cases (166). The occurrence of mutations in the TNNC1 gene is roughly 1% (167). The frequency of TNNT2 mutations in DCM is around 3% (168). Patients with TNNC1 gene mutations are typically diagnosed at a younger age and have a higher risk of experiencing potentially fatal events, which often manifest as early severe systolic heart failure, necessitating heart transplant surgery (163, 169). Research indicates that a reduced sensitivity of myofilaments to Ca2+ plays a critical role in the pathophysiology of filament-associated DCM. Enhancing myofilament sensitivity to Ca2+ in the early stages of DCM might be an effective treatment strategy (170). Xin actin-binding repeat containing proteins (XIRPs) are a group of rhabdom-specific proteins. The XIN protein, encoded by the XIRP1 gene and also known as HXin-α or CMYA1, is a rhabdom-specific gene in the XIRP family. Overexpression of the repeating isomer XINB can ameliorate DCM remodeling induced by TNNT2-ΔK210 mutations in mice, partially reversing cardiac dilation, systolic dysfunction, and cardiac fibrosis. Therefore, XIN could be a potential therapeutic target (171).

## 6. Conclusion

This review systematically summarizes the genes and mechanisms implicated in dilated cardiomyopathy, as well as the latest research directions in understanding its causes. It should be noted that with the advancement of medical technology, the diagnosis rate of dilated cardiomyopathy has been increasing. Nonetheless, patients often present with early onset, severe clinical manifestations, and poor prognosis. The standard approach for preventing or treating heart failure is currently the first-line treatment for patients with dilated cardiomyopathy. Cardiac resynchronization therapy and implantable cardioverter-defibrillators may be necessary to prevent life-threatening arrhythmias. It is recommended that all patients with dilated cardiomyopathy undergo sequencing of known cardiomyopathy genes. Gene-level therapy may represent a new approach for future treatments, although our current understanding of disease pathogenesis and gene therapy is primarily derived from preclinical animal models. This review also has some limitations, primarily that it only encompasses genes with substantial supporting evidence within the realm of evidence-based medicine. Due to the constraints of the review's length, there is a limited number of genes currently being researched and a lack of supporting experimental data. Consequently, some genes pertinent to “moderate classification” and “limited classification” have not been included in this review. Further research in this area is warranted.
