# Supplementary material for: Comprehensive review on gene mutations contributing to dilated cardiomyopathy
Source: Front Cardiovasc Med. 2023 Dec 1;10:1296389. doi: 10.3389/fcvm.2023.1296389 (PMC10722203; doi:10.3389/fcvm.2023.1296389)
Supplement: Supplementary file 1 [file Table1.docx]

| Causes of dilated cardiomyopathy | |
| --- | --- |
| Acquired | Genetic |
| PPCM | Ion channel |
| Deposition | Nucleus |
| Neuromuscular | Nuclear envelope |
| Infections | Sarcomere |
| Inflammation/autoimmune | Cytoskeleton |
| Drugs/toxic |  |
| Tachycardia induced |  |

Diagnosis of dilated cardiomyopathy

| Basic | Individual |
| --- | --- |
| ECG | Holter ECG |
| Echocardiography | Coronary angiography |
| Blood sample | CMR |
| Medical history | Genetic analysis |
| Family history | Scintigraphy |
|  | EMB |

PPCM, Peripartum cardiomyopathy; CMR, cardiovascular magnetic resonance imaging; EMB, Endomyocardial biopsy;
