# Supplementary material for: Comprehensive review on gene mutations contributing to dilated cardiomyopathy
Source: Front Cardiovasc Med. 2023 Dec 1;10:1296389. doi: 10.3389/fcvm.2023.1296389 (PMC10722203; doi:10.3389/fcvm.2023.1296389)
Supplement: Supplementary file 2 [file Table2.docx]

| **Gene** | **Protein** | **Estimated**  **contribution to**  **total number**  **of patients** | **Related mechanisms that trigger DCM** | **clinical manifestation** |
| --- | --- | --- | --- | --- |
| TTN | Titin | 25% | Haploinsufficiency;  poison peptide/dominant negative mechanism; Disruption of cardiac metabolism and signaling, and loss of function. | Arrhythmias(atrial fibrillation and ventricular arrhythmias) |
| LMNA | Lamin A/C | 6% | Mechanical hypotheses; Gene expression hypotheses; Cytotoxicity hypotheses | Conduction system disorders (sinus bradycardia, sinus node arrest with junctional rhythm, or heart block); arrhythmias (chronic atrial fibrillation/supraventricular arrhythmias/ventricular arrhythmias) |
| DSP | Desmoplakin | NA | Inflammation | Palmoplantar keratoderma; Fatal ventricular arrhythmia; Acute myocardial injury; |
| DES | Desmin | <1.6% | Affects the structure of the heart; Affects myocardial metabolism of glucose, fatty acids and amino acids | Neurologic symptoms (myopathy or muscle weakness); cardiac symptoms (heart block: Atrioventricular block, arrhythmia: atrial fibrillation); respiratory insufficiency |
| MYH7 | Myosin 7 | 1-5.3% | Damage the structural or functional integrity of the myotome | Atrial fibrillation; Atrial fibrillation |
| BAG3 | BAG family molecular chaperone regulator 3 | NA | cellular senescence | progressive heart failure |
| FLNC | Filamin C | NA | Activation of platelet-derived growth factor receptor-α pathway | left ventricular dilatation with systolic dysfunction；myocardial fibrosis |
| PLN | Cardiac phospholamban | <1% | Defects in calcium metabolism | early ventricular arrhythmia； end-stage heart failure |
| RBM20 | RNA-binding protein 20 | 3% | Alters expression of protein subtypes that maintain muscle structure and heart function; | Severe impairment of cardiac systolic function; Cardiac arrhythmias (sustained ventricular arrhythmias/sudden cardiac death/atrial fibrillation) |
| SCN5A | Sodium channel protein type 5 subunit α | 2–3% | Loss of functionality in the cardiac sodium channel; Disruption of the interaction between SCN5A and other constituents, leading to structural alterations and impairment of contraction. | Arrhythmias (atrial fibrillation/ventricular tachycardia); Heart block. |
| TNNC1 | Troponin C, slow skeletal and cardiac muscles | <1% | Change its binding affinity for Ca2+ or by altering the interaction of cTnC with its binding partner; reduced sensitivity of myofilaments to Ca2+ | Early severe systolic heart failure |
| TNNT2 | Troponin T, cardiac muscle | 3% | XIN deficiency | cardiac dilation；systolic dysfunction； cardiac fibrosis； |
